# Aldosterone-to-renin ratio is related to arterial stiffness when the screening criteria of primary aldosteronism are not met

**DOI:** 10.1038/s41598-020-76718-7

**Published:** 2020-11-13

**Authors:** Eeva Kokko, Pasi I. Nevalainen, Manoj Kumar Choudhary, Jenni Koskela, Antti Tikkakoski, Heini Huhtala, Onni Niemelä, Marianna Viukari, Jukka Mustonen, Niina Matikainen, Ilkka Pörsti

**Affiliations:** 1 Faculty of Medicine and Health Technology, Tampere University, Tampere, Finland; 2 Department of Internal Medicine, Tampere University Hospital, Tampere, Finland; 3 Department of Clinical Physiology, Tampere University Hospital, Tampere, Finland; 4 Faculty of Social Sciences, Tampere University, Tampere, Finland; 5 Clinical Laboratory and Medical Research Unit, Seinäjoki Central Hospital, Seinäjoki, Finland; 6 Endocrinology, Helsinki University Hospital and Research Programs Unit, Clinical and Molecular Medicine, University of Helsinki, Helsinki, Finland

**Keywords:** Endocrinology, Endocrine system and metabolic diseases, Physiology, Circulation

## Abstract

Aldosterone-to-renin ratio (ARR) is a screening tool for primary aldosteronism (PA), but the significance of ARR when the PA criteria are not met remains largely unknown. In this cross-sectional study we investigated the association of ARR with haemodynamic variables in 545 normotensive and never-medicated hypertensive subjects (267 men, 278 women, age range 19–72 years) without suspicion of PA. Supine haemodynamic data was recorded using whole-body impedance cardiography and radial tonometric pulse wave analysis. In sex-adjusted quartiles of ARR, determined as serum aldosterone to plasma renin activity ratio, the mean values were 282, 504, 744 and 1467 pmol/µg of angiotensin I/h, respectively. The only difference in haemodynamic variables between the ARR quartiles was higher pulse wave velocity (PWV) in the highest quartile versus other quartiles (*p* = 0.004), while no differences in blood pressure (BP), heart rate, wave reflections, cardiac output or systemic vascular resistance were observed between the quartiles. In linear regression analysis with stepwise elimination, ARR was an independent explanatory factor for PWV (β = 0.146, *p* < 0.001, R^2^ of the model 0.634). In conclusion, ARR was directly and independently associated with large arterial stiffness in individuals without clinical suspicion of PA. Therefore, ARR could serve as a clinical marker of cardiovascular risk.

Trial registration: ClinicalTrails.gov: NCT01742702.

## Introduction

Primary aldosteronism (PA) is the most common form of secondary hypertension^1^, yet most cases remain undiagnosed^2,3^. The estimated prevalence of PA has conventionally ranged from 5 to 15% among hypertensive patients^4–6^, but according to a recent study, the prevalence might be as high as 16% to 22%^7^. It is known that PA is associated with significantly higher risk of cardiovascular events and target organ damage than essential hypertension at comparable levels of blood pressure^5,8,9^. Aldosterone excess increases the accumulation of growth factors and collagen fibres in arterial wall^10,11^ and it has been shown that PA patients have an increased arterial wall stiffness in comparison with patients who have essential hypertension^12,13^.

Screening of PA is based on the aldosterone-to-renin ratio (ARR). The Endocrine Society recommends the screening in the following cases with clinical suspicion pf PA: Blood pressure > 150/100 mmHg on three different days, drug resistant hypertension that is uncontrolled with ≥ three antihypertensive drugs, controlled hypertension with ≥ four antihypertensive drugs, hypertension with spontaneous or diuretic-induced hypokalaemia, hypertension with adrenal incidentaloma, hypertension with a family history of hypertension or cerebrovascular events at a young age (< 40 years), hypertension together with sleep apnoea, and all first-degree relatives of patients with PA^1,5^.

The role of ARR for screening of PA is robust, but there is a scarcity of data evaluating the significance of ARR in subjects who remain below the screening threshold for PA. Previously, ARR has been associated with the development and severity of hypertension, even in patients without excessive aldosterone levels^14–18^. A Japanese long-term observational analysis demonstrated that elevated ARR is associated with an increased incidence of cardiovascular disease in patients with essential hypertension^19^. In 60 healthy adults with a mean age 43 years, Shapiro et al. reported a direct correlation (r_P_ = 0.298) between ARR and pulse wave velocity (PWV)^20^. In 2000 participants of the Framingham study, Lieb et al. found that ARR was a significant independent correlate of PWV (β = 0.20). However, the participants were not screened for the presence of PA, 33% were ingesting antihypertensive medications, and 12% had diabetes mellitus^18^.

In this cross-sectional study we analysed the association of ARR with several cardiovascular variables in normotensive subjects and previously undiagnosed never-medicated hypertensive patients. We tested the hypothesis whether ARR correlates with haemodynamic parameters in subjects who are not fulfilling the clinical criteria for the screening of PA.

## Methods

### Study subjects

The recruitment of the study subjects has previously been described in detail^21–23^. All subjects were examined by a physician and routine laboratory analyses for elevated blood pressure (BP) were taken^24^. The medical history, lifestyle behaviour and family history were documented. Alcohol use was evaluated as standard drinks (~ 12 g of absolute alcohol) per week, and smoking amount was estimated in pack-years. The exclusion criteria were history of coronary artery disease, stroke, cardiac insufficiency, valvular heart disease, chronic kidney disease^25^, secondary hypertension, alcohol or substance abuse^26^, psychiatric illnesses other than mild to moderate depression or anxiety, heart rhythm other than sinus rhythm, use of antihypertensive or uric-acid-lowering medications, and ongoing pregnancy. Altogether 606 subjects, aged 19–72 years, were eligible for the study. Data of ARR was missing in 48 subjects while 13 subjects met the screening criteria of PA (serum aldosterone > 550 pmol/l and ARR > 750 pmol/µg of angiotensin I/h, plasma potassium concentration < 3.3 mmol/l, BP > 150/100 mmHg) and were therefore excluded. The Endocrine Society screening for PA is based on BP > 150/100 mmHg on three different days^1,5^. In the present study the BP criteria were applied on the 5-min laboratory BP measurements (see below), and the results were confirmed by applying the criteria for the office BP measurements on a single occasion. The final study population consisted of 545 subjects.

Signed informed consent was obtained from all participants. The study complies with the declaration of Helsinki and was approved by the Ethics Committee of the Pirkanmaa Hospital District (study code R06086M).

### Laboratory analyses and indexes glucose tolerance

Blood and urine samples were taken after 12 h of fasting. Plasma renin activity (GammaCoat Plasma Renin Activity 125-I RIA Kit, DiaSorin, Saluggia, Italy) and aldosterone concentration (Active Aldosterone RIA, Beckman Coulter, Fullerton, CA, USA) were determined using commercial kits. Plasma C-reactive protein (CRP), sodium, potassium, glucose, creatinine, cystatin C, uric acid, triglyceride, and total, HDL and LDL (high- and low-density lipoprotein, respectively) cholesterol concentrations were determined using Cobas Integra 800 (F. Hoffmann-LaRoche Ltd, Basel, Switzerland). Insulin and parathyroid hormone (PTH) were determined using electrochemiluminescence immunoassay (Cobas e411, Roche Diagnostics). Estimated glomerular filtration rate (eGFR) was calculated using the Chronic Kidney Disease – Epidemiology collaboration (CKD-EPI) cystatin C equation^27^. Insulin sensitivity was evaluated by the quantitative insulin sensitivity check index (QUICKI)^28^, and homeostatic model assessment of insulin resistance (HOMA-IR)^29^. In addition, the subjects were invited to participate in a standard 75-g oral glucose tolerance test (OGTT) for the determination of the Matsuda index^30^.

### Experimental protocol

Haemodynamic recordings were performed in a noiseless, temperature-controlled laboratory by research nurses^21,22,31^. Products containing caffeine, smoking or heavy meal were not allowed for ≥ 4 h, and alcohol consumption was not allowed for ≥ 24 h before the investigation. The subjects rested supine on the examination table with impedance cardiography electrodes placed on body surface, tonometric sensor for pulse wave analysis on left radial pulsation, and oscillometric brachial cuff for BP calibration to the right upper arm. The left arm with the tonometric sensor was stabilized to 90° in a support, which held the measurement probe at the heart level.

The measurement consisted of a 5-min period, during which haemodynamic data was captured continuously. For the statistical analyses, the mean values of each 1-min period of recording were calculated. The repeatability and reproducibility of the protocol have been demonstrated to be good^21,22,31^.

### Pulse wave analysis

Radial BP and pulse wave were recorded by a tonometric sensor (Colin BP-508 T, Colin Medical Instruments Corp., USA) fixed on the radial pulse^21,22^. The radial BP signal was calibrated approximately every 2.5 min by brachial BP measurements. Aortic BP was derived using the SphygmoCor pulse wave monitoring system (SphygmoCor PWMx, AtCor medical, Australia)^32^. Aortic pulse pressure, augmentation index (AIx, augmented pressure/pulse pressure * 100), and AIx adjusted to heart rate 75/min (AIx@75) were also determined. The central forward wave amplitude was defined as the difference between waveform foot and first systolic inflection point pressure in the aortic waveform^33,34^.

### Whole-body impedance cardiography

We used the CircMon device (JR Medical Ltd., Tallinn, Estonia) to assess changes in body electrical impedance during cardiac cycles to record heart rate, stroke volume, cardiac output, and PWV^35–37^. Systemic vascular resistance was calculated from radial BP and cardiac output measured by CircMon. Stroke volume, cardiac output and systemic vascular resistance were presented as indexes related to body surface area calculated using the DuBois equation^38^. The method and electrode configuration has been previously reported^36,37^.

With CircMon the recorded stroke volume and cardiac output are in good agreement with values obtained utilizing 3-dimensional echocardiography^31^ and the thermodilution and direct oxygen Fick methods^35,36^, and the PWV values show very good correlation with values measured using ultrasound or the tonometric method^37,39^.

### Statistics

To illustrate the influence of ARR on the haemodynamic variables, the study participants were divided into quartiles of ARR. As serum aldosterone concentration was slightly higher in women than in men (see ‘Results’), the ARR quartiles 1–4 were constructed separately for women and men.

Analyses of normally distributed data were performed using analysis of variance (ANOVA) and ANOVA for repeated measurements, and non-normally distributed data using Kruskal–Wallis test with Mann–Whitney U-test in the post-hoc analyses. The Bonferroni correction was applied in all post-hoc analyses. The ANOVA for repeated measures analyses were adjusted for sex, age, BMI, and eGFR, as appropriate, by using these variables as covariates in the analyses. Normally distributed variables in the tables were presented as means and standard deviations and non-normally distributed variables as medians and 25th–75th percentiles. The figures were depicted as means and standard errors of the mean (SEM). Pearson’s correlations were calculated for normally distributed and Spearman's correlations for non-normally distributed variables. The values of the haemodynamic variables for the statistics in Table 1 and regression analysis were calculated as averages from the minutes 3–5 of the recordings when the signal was most stable. *P* < 0.05 was considered significant.

**Table 1:** Results of the study participants in sex adjusted quartiles of aldosterone-to-renin ratio: demographic data and clinical characteristics.

	Quartile 1 (n = 135)	Quartile 2 (n = 137)	Quartile 3 (n = 137)	Quartile 4 (n = 136)
Male/female (n)	66/69	67/70	67/70	67/69
Number of female hormone users	16	12	21	21
Number of oestrogen users	11	10	18	16
Age (years)	41.9 (11.4)	44.8 (11.5)	46.0 (11.5)*	49.5 (10.9)*†
Weight (kg)	78.7 (15.8)	79.6 (15.6)	80.0 (14.8)	81.8 (15.0)
Height (cm)	173.2 (9.5)	172.2 (9.6)	173.8 (8.8)	172.7 (9.2)
Body mass index (kg/m^2^)	26.1 (4.2)	26.8 (4.6)	26.4 (4.1)	27.3 (3.9)
Alcohol (standard drinks/week)	3 [0–5]	3 [1–6]	3 [1–6]	2 [0–4]
Current smokers (n)	22	18	11	13
**Office measurements**^**a**^				
Systolic BP (mmHg)	135.3 (20.0)	137.5 (20.2)	139.7 (18.6)	144.1 (21.0)*
Diastolic BP (mmHg)	86.2 (13.1)	87.6 (12.2)	88.2 (10.7)	91.8 (11.6)*†
Heart rate (bpm)	67.5 (9.6)	67.0 (9.3)	65.2 (9.6)	67.8 (9.1)
Normotensive/hypertensive (n)	75 / 60	71 / 66	67 / 70	49 / 87*
**Haemodynamic measurements**^**b**^				
Systolic BP (mmHg)	127.4 (18.1)	129.0 (18.1)	129.1 (17.8)	132.0 (18.3)
Diastolic BP (mmHg)	72.5 (11.9)	73.4 (12.0)	73.4 (12.0)	76.1 (12.8)
Heart rate (bpm)	63.9 (10.2)	63.8 (10.3)	62.1 (9.3)	63.6 (8.1)
Normotensive/hypertensive (n)	107 / 28	103 / 34	100 / 37	95 / 41

BP, blood pressure; mean (standard deviation) or median [25th–75th percentile].

*P < 0.05 versus Q1; †*P* < 0.05 versus Q2.

^a^Office BP was available from 123–129 participants per group.

^b^Haemodynamic measurement systolic/diastolic BP values were 12.7–15.6/16.0–17.3 mmHg, and heart rate 2.8–4.5 beats/min, lower than in the office with no significant differences between the quartiles.

To investigate factors independently associated with PWV in the whole study population, linear regression analysis with stepwise elimination was applied. The covariates in the analysis were age, sex, BMI, categorised smoking status (never, present, previous), categorised alcohol consumption (low, moderate, high); plasma aldosterone, renin, aldosterone-to-renin ratio, HDL cholesterol, LDL cholesterol, triglycerides, CRP, uric acid, calcitriol, PTH, QUICKI, eGFR, ejection duration, heart rate, and mean aortic pressure. Coefficients (B) and standard coefficients (Beta) of regression were calculated, and assumptions of linearity were confirmed by the analysis of residuals. IBM SPSS Statistics Version 26 (IBM Corporation, Armonk, NY, USA) was used for statistics.

## Results

### Study population and laboratory values

The proportion of male subjects was 49%. Plasma renin activity and ARR did not significantly differ between women versus men [0.99 (0.90) vs. 0.97 (0.95) ng of angiotensin I/ml/h; 779 (527) vs. 719 (531) pmol/µg of angiotensin I/h, respectively; mean (SD)]. However, serum aldosterone concentration was higher in women than in men (561 (481) vs. 449 (208) pmol/l, respectively, *p* < 0.001). Therefore, the ARR results were examined in sex-adjusted quartiles (Q) (Table 1).

None of the subjects used anti-hypertensive medicines. The proportions of female hormone users did not differ between the ARR quartiles. Mean participant age was 46 years, with an age range of 19–72 years in male and 21–72 years in female subjects. Participant age was higher in Q3 versus Q1, and in Q4 versus Q1 and Q2. Mean BMI was 26.6 kg/m^2^ with no differences between the quartiles. Average alcohol use was moderate 4.3 standard drinks/week, and the prevalence of present and previous smokers was 12% and 31%, respectively. Alcohol intake and the prevalence of smokers were corresponding in all quartiles (Table 1).

When applying the office cut-off values of hypertension (systolic BP ≥ 140 or diastolic BP ≥ 90)^24^, the number of never-medicated hypertensive subjects was 283 (51.9%). Office systolic BP was higher in Q4 than in Q1, and office diastolic BP in Q4 than in Q1 and Q2. The proportion of hypertensive subjects in the office measurements was higher in Q4 than in Q1.

During the haemodynamic measurements, systolic and diastolic BP values were 13–16 and 16–17 mmHg lower, respectively, and heart rate 3–5 beats/min lower, than in the office measurements, with no significant differences between the quartiles. With the office cut-off values of hypertension, no significant differences were found in the proportion of hypertensive subjects between the quartiles during the haemodynamics measurements (Table 1).

In sex adjusted quartiles of ARR, the mean values were 282, 504, 744 and 1467 pmol/µg of angiotensin I/h, respectively (Table 2). The differences in ARR were explained by variations in plasma renin activity, as aldosterone concentrations did not differ between the quartiles. Plasma renin activity was different in all quartiles with highest values in Q1 and lowest values in Q4. In our study, age was inversely correlated both with plasma renin activity and serum aldosterone concentration (r_S_ =  − 0.369 and r_S_ =  − 0.253, respectively, *p* < 0.01 for both), while age and ARR were directly correlated (r_S_ = 0.238, *p* < 0.01).

**Table 2:** Laboratory results of the study participants in sex adjusted quartiles of aldosterone-to-renin ratio.

	Quartile 1 (n = 135)	Quartile 2 (n = 137)	Quartile 3 (n = 137)	Quartile 4 (n = 136)
Aldosterone-to-renin ratio (pmol/µg of Ang I/h)	287 [227–342]	497 [451–560]*	740 [674–799]*†	1302 [1085–1614]*†‡
Serum aldosterone (pmol/l)	419 [297–552]	448 [324–599]	431 [336–636]	427 [323–575]
Plasma renin activity (ng of Ang I/ml/h)	1.47 [1.12–2.17]	0.89 [0.67–1.21]*	0.58 [0.46–0.88]*†	0.29 [0.20–0.44]*†‡
**Sodium (mmol/l)**	140.4 (1.8)	139.9 (1.8)	140.1 (2.1)	140.7 (1.9)†
24-h urine sodium excretion (mmol)^a^	153 (62)	155 (61)	154 (65)	148 (57)
**Potassium (mmol/l)**	3.8 (0.2)	3.8 (0.3)	3.8 (0.3)	3.8 (0.3)
24-h urine potassium excretion (mmol)^a^	85 (30)	87 (30)	86 (27)	79 (22)
Calcium (mmol/l)	2.30 (0.09)	2.29 (0.10)	2.30 (0.10)	2.29 (0.10)
Parathyroid hormone (pmol/l)	4.37 (1.59)	4.37 (1.41)	4.43 (1.49)	4.71 (1.59)
25OH-D_3_ (nmol/l)	74.2 (33.5)	66.1 (28.3)	74.9 (45.4)	70.7 (30.0)
1,25(OH)_2_-D_3_ (pmol/l)	110.2 (31.4)	109.3 (36.3)	107.6 (33.8)	106.7 (34.9)
C-reactive protein (mg/l)	0.7 [0.5–1.5]	1.0 [0.5–2.2]	0.5 [0.4–1.3]†	1.0 [0.5–2.1]‡
Creatinine (µmol/l)	74 (13)	72 (13)	75 (14)	73 (14)
Cystatin C	0.81 (0.14)	0.83 (0.14)	0.85 (0.15)	0.86 (0.15)
Estimated GFR (ml/min/1.73 m^2^)	104.7 (18.1)	100.9 (17.8)	98.3 (18.0)*	95.3 (17.1)*
Total cholesterol (mmol/l)	5.0 (1.0)	5.1 (1.0)	5.0 (1.0)	5.3 (1.0)*
Triglycerides (mmol/l)	1.11 [0.67–1.38]	1.22 [0.74–1.43]	1.21 [0.67–1.46]	1.27 [0.87–1.54]
HDL cholesterol (mmol/l)	1.6 (0.4)	1.6 (0.5)	1.6 (0.4)	1.5 (0.4)
LDL cholesterol (mmol/l)	2.9 (0.9)	3.0 (1.0)	2.9 (0.9)	3.2 (0.9)
Uric acid (µmol/l)	302 (71)	297 (65)	292 (71)	303 (86)
Glucose (mmol/l)	5.3 (0.5)	5.5 (0.5)	5.4 (0.5)	5.6 (0.8)*
Insulin (mU/l)	6.8 [5.0–10.4]	6.8 [5.3–9.7]	5.9 [4.3–8.1]	7.0 [4.7–9.4]
Matsuda index^b^	5.74 [3.74–8.18]	5.94 [3.50–8.78]	7.62 [4.44–9.69]	5.80 [3.89–7.72]
HOMA-IR	1.56 [1.14–2.61]	1.70 [1.23–2.37]	1.43 [1.02–2.11]	1.69 [1.13–2.40]
QUICKI	0.357 [0.331–0.375]	0.352 [0.335–0.371]	0.362 [0.341–0.382]	0.353 [0.335–0.376]

Results shown as mean (standard deviation) or median [25th–75th percentile]; GFR, estimated glomerular filtration rate from plasma cystatin C using the CKD-EPI formula^27^.

HDL, high-density lipoprotein; LDL, low-density lipoprotein; HOMA-IR, homeostatic model assessment of insulin resistance; QUICKI, quantitative insulin sensitivity check index.

*P < 0.05 versus Q1; †*P* < 0.05 versus Q2; ‡*P* < 0.05 versus Q3.

^a^n = 101–118 for 24-h urine excretion results.

^b^n = 88–98 for Matsuda index results in each quartile.

Average measures of plasma electrolytes, PTH, CRP, uric acid, glucose metabolism and renal function were within the normal range in all quartiles (Table 2). In seven participants fasting plasma glucose ranged 7.0–10.3 mmol/l, i.e. in the diabetic range, but none of them presented with glucosuria. Altogether 53 participants had impaired fasting plasma glucose concentration ranging 6.2–6.9 mmol/l. Plasma sodium concentration was minimally higher in Q4 than in Q2. No differences were observed in 24-h excretion of sodium or potassium to the urine, plasma potassium, calcium, PTH, creatinine, uric acid, triglycerides, HDL cholesterol or LDL cholesterol. However, total cholesterol was somewhat higher in Q4 when compared to Q1. Estimated GFR was lower in Q3 and Q4 when compared to Q1. Although fasting plasma glucose concentration was higher in Q4 than in Q1, all the evaluated insulin sensitivity indices (QUICKI, HOMA-IR, Matsuda index) were invariable between the ARR quartiles (Table 2).

### Haemodynamic measurements

**Figure 1:**
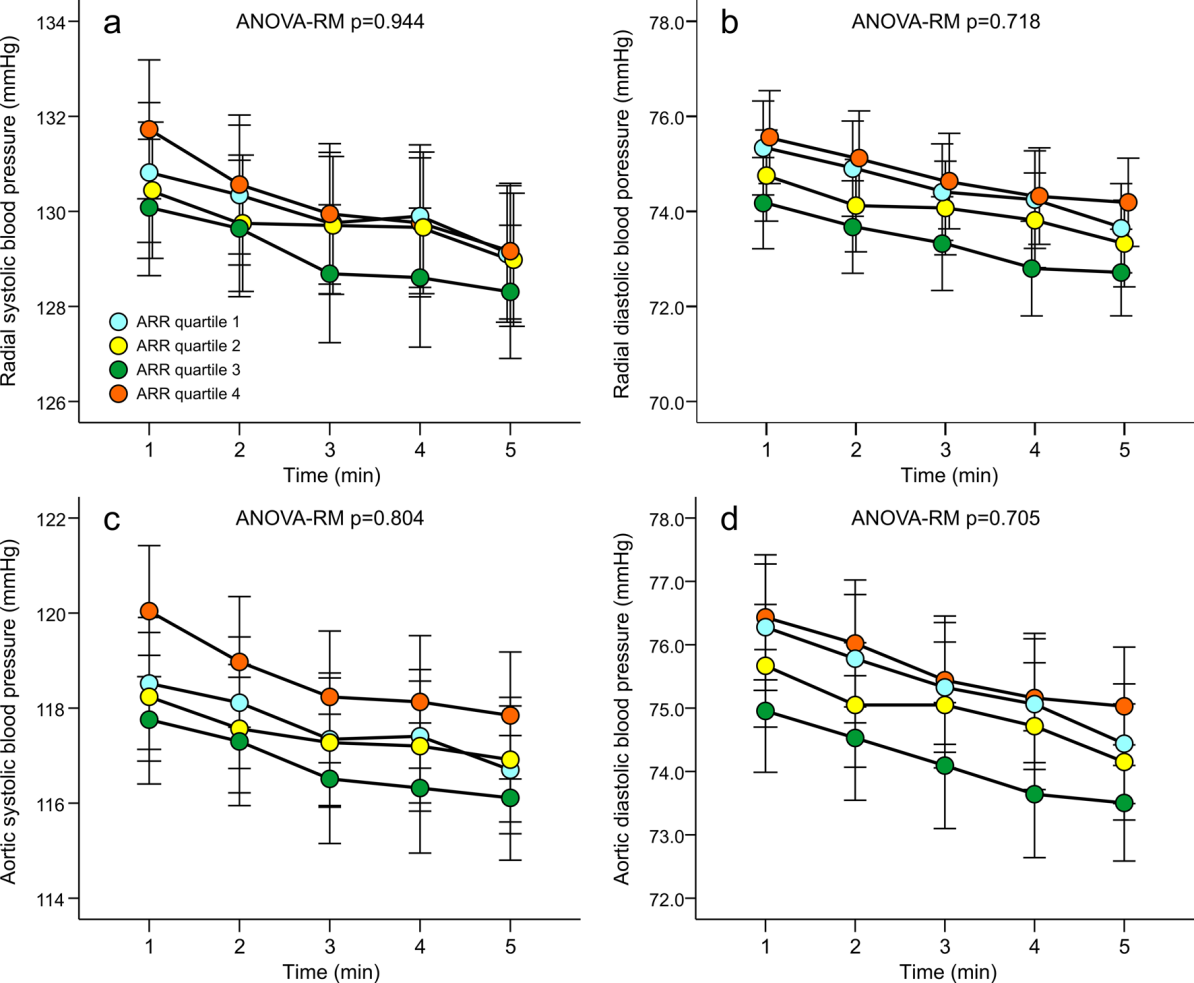
Radial systolic (**a**) and diastolic (**b**) blood pressure calibrated from brachial blood pressure measurements, and aortic systolic (**c**) and diastolic (**d**) blood pressure in quartiles (n = 135–137) of aldosterone-to-renin ratio (ARR); analyses were adjusted for sex, age, and estimated glomerular filtration rate; ANOVA-RM, analysis of variance for repeated measurements, results are depicted as mean and standard error of the mean.

**Figure 2:**
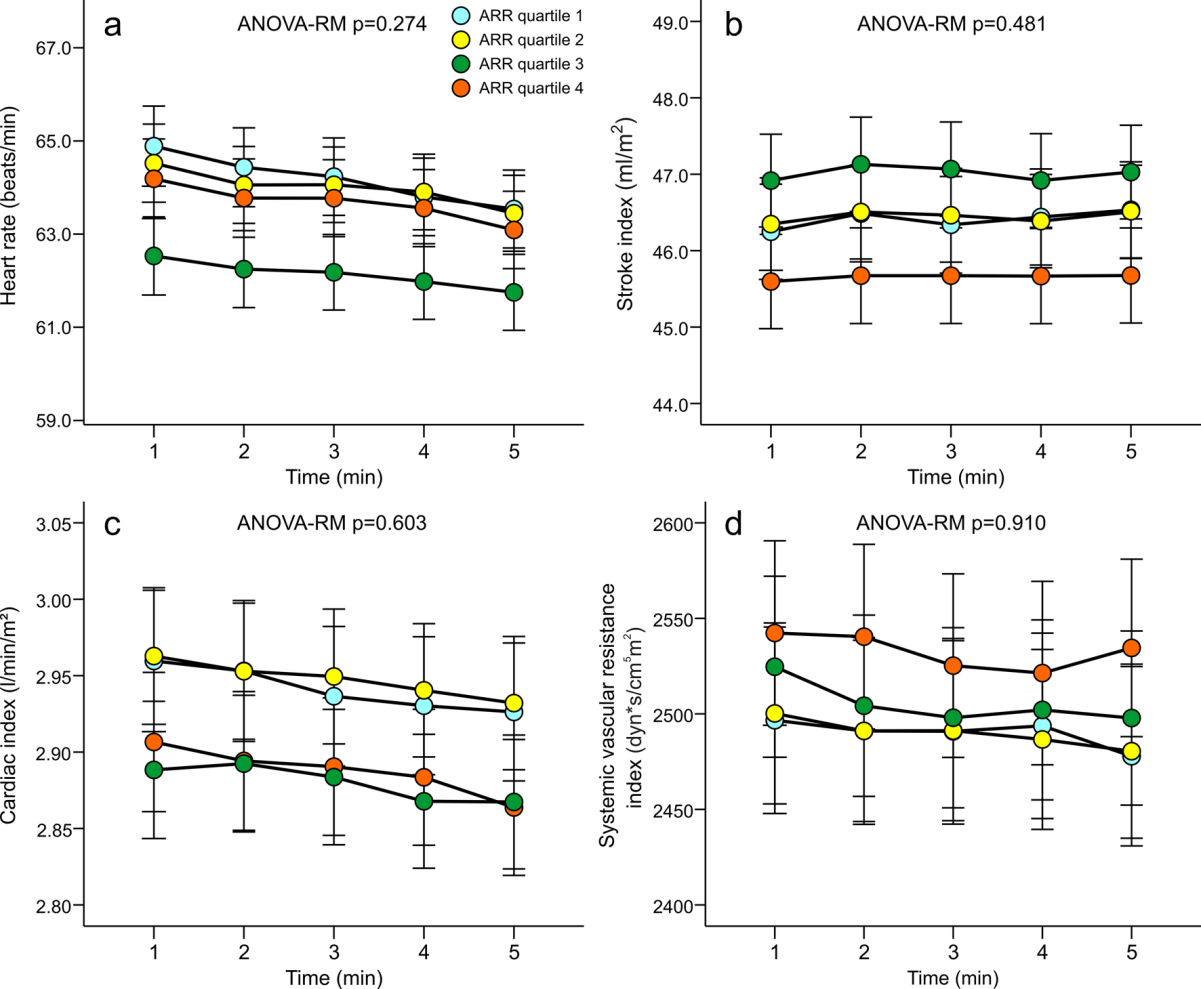
Heart rate (**a**), stroke index (**b**), cardiac index (**c**) and systemic vascular resistance index (**d**) in quartiles (n = 135–137) of aldosterone-to-renin ratio (ARR); analyses were adjusted for sex, age, and estimated glomerular filtration rate; ANOVA-RM, analysis of variance for repeated measurements, mean and standard error of the mean.

**Figure 3:**
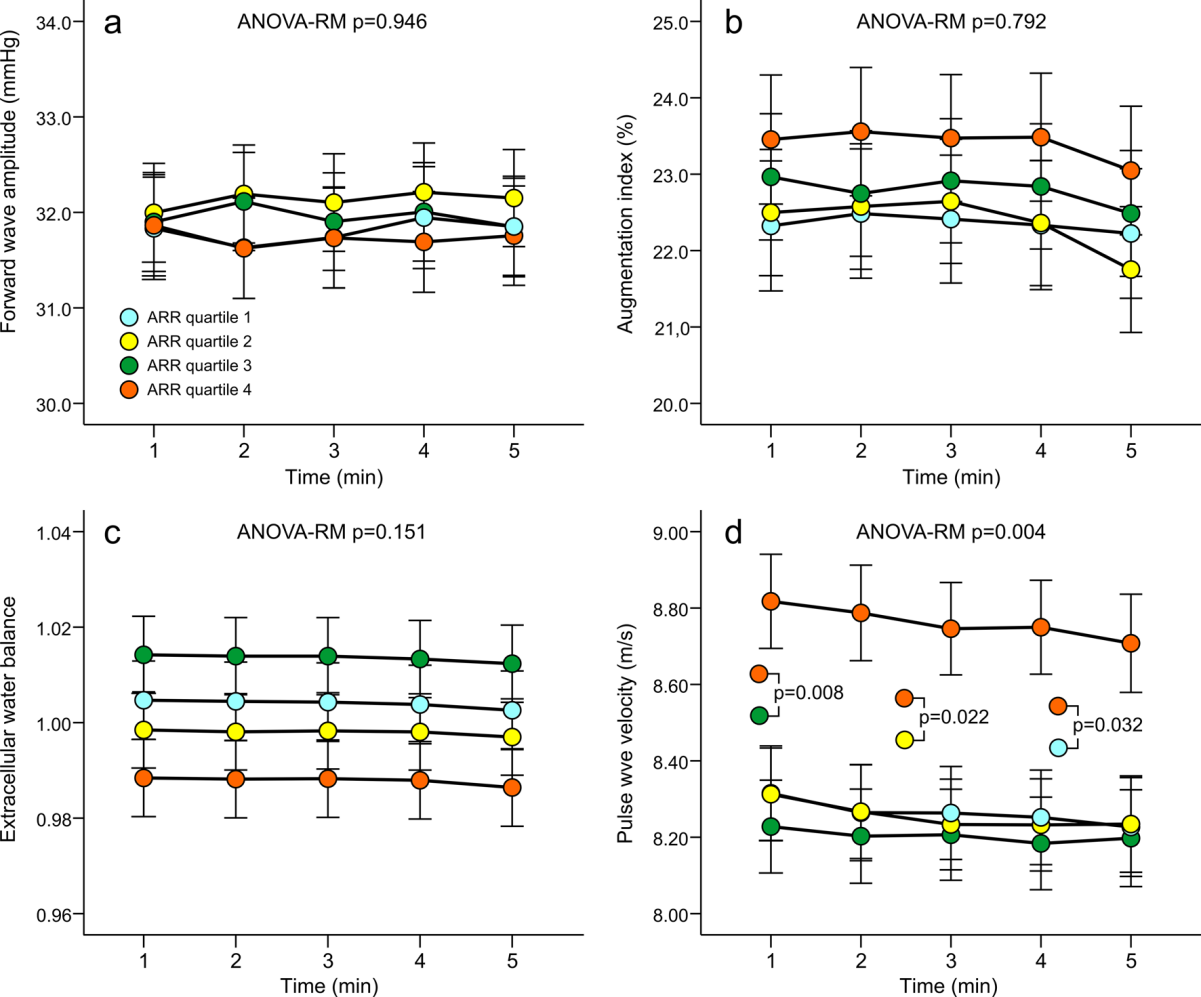
Forward wave amplitude (**a**), augmentation index (**b**), extracellular water balance (**c**) and aortic-to-popliteal pulse wave velocity (**d**) in quartiles (n = 135–137) of aldosterone-to-renin ratio (ARR); analyses were adjusted for sex, age, and estimated glomerular filtration rate; ANOVA-RM, analysis of variance for repeated measurements, mean and standard error of the mean.

*ARR and haemodynamics*. The results representing the haemodynamic variables in the sex-, age-, and eGFR-adjusted quartiles of ARR are shown in Figs. 1, 2 and 3. No differences in radial or aortic BP were observed between the quartiles (Fig. 1). Heart rate, stroke index, cardiac index and systemic vascular resistance index were also similar in all quartiles (Fig. 2). No differences were found in forward wave amplitude, augmentation index or extracellular water balance between the quartiles, either. However, in the absence of differences in central BP, aortic to popliteal PWV was clearly higher in Q4 versus other quartiles (*p* = 0.004) (Fig. 3). Among the study subjects, the Spearman correlation between ARR and PWV was 0.219 (*p* < 0.01).

*Aldosterone and haemodynamics*. When examined in sex-adjusted quartiles of serum aldosterone concentration, the quartiles presented with differences in age and BMI (not shown). In the sex-, age- and BMI-adjusted quartiles of serum aldosterone concentration, no differences in the haemodynamic variables were detected between the quartiles except for PWV, the value of which was lower in Q1 versus Q4 (8.12 ± 0.12 vs. 8.58 ± 0.12 m/s, *p* = 0.045).

*Renin activity and haemodynamics*. In sex-adjusted quartiles of plasma renin activity, no additional adjustments were needed (not shown). Corresponding to the results in quartiles of aldosterone concentration, all haemodynamic variables in quartiles of renin activity were similar except for PWV. However, the difference in PWV was observed between Q1 and Q2 (8.65 ± 0.13 vs. 8.08 ± 0.12 m/s, *p* = 0.006), while PWV in Q1, Q3 and Q4 was corresponding (ranging from 8.37 ± 0.12 to 8.65 ± 0.12 m/s, *p* = 0.559–1.000).

### Determinants of large arterial stiffness

The results of the linear regression analyses with stepwise elimination are presented in Table 3. The independent explanatory factors for PWV were age, ejection duration, uric acid concentration, mean aortic pressure, ARR, BMI, low alcohol consumption, and heart rate (R^2^ = 0.634). The variables in the model included both aldosterone concentration and renin activity, yet neither of them turned out as independent predictors for PWV. QUICKI was used as an insulin sensitivity variable in the model, as the Matsuda index was not available from all participants (Table 2). In our study population the correlation between QUICKI and Matsuda index was 0.884 (*p* < 0.001) and between QUICKI and HOMA-IR-0.720 (*p* < 0.001).

**Table 3:** Significant explanatory variables for aortic to popliteal pulse wave velocity in linear regression analysis with stepwise elimination.

Pulse wave velocity (m/s)	B	Beta	*P*
**R**^**2**^ = **0.634**			
(constant)	5.046		
Age	0.071	0.488	< 0.001
Ejection duration	− 0.016	− 0.185	< 0.001
Uric acid	0.005	0.199	< 0.001
Mean aortic pressure	0.018	0.148	< 0.001
Aldosterone-to-renin ratio	4.68 × 10^–4^	0.146	< 0.001
Body mass index	0.041	0.103	0.001
Heart rate	0.017	0.098	0.010
Low alcohol consumption category	− 0.220	− 0.062	0.025

Variables included in the model correlated with pulse wave velocity with a *p*-value < 0.05: age, sex, BMI, categorised smoking status, categorised alcohol consumption; plasma aldosterone, renin, aldosterone-to-renin ratio, HDL cholesterol, LDL cholesterol, triglycerides, CRP, uric acid, calcitriol, PTH; QUICKI, eGFR, ejection duration, heart rate, and mean aortic pressure. HDL, high-density lipoprotein; LDL, low-density lipoprotein; QUICKI, quantitative insulin sensitivity check index; eGFR, estimated glomerular filtration rate from plasma cystatin C using the CKD-EPI formula^27^.

When the office cut-off for hypertension (BP ≥ 140/90 mmHg) was applied for the laboratory measurements^24^, ARR was an independent explanatory factor for PWV in both the normotensive (*p* < 0.001, R^2^ = 0.580) and hypertensive (*p* = 0.012, R^2^ = 0.523) subgroups. The result did not change when applying the home BP measurement cut-off (BP ≥ 135/85 mmHg).

If the office BP measurements were applied for the exclusion of participants with potential PA, 499 subjects were eligible for the statistical analyses. Also, in this analysis ARR was an independent explanatory factor for PWV (*p* < 0.001), while the other explanatory factors were age, mean aortic pressure, heart rate, and plasma concentrations of HDL cholesterol, uric acid, and triglycerides (R^2^ = 0.572 of the model).

## Discussion

In this study we investigated the association of ARR with several cardiovascular variables in normotensive subjects and never-medicated hypertensive patients without clinical suspicion of PA and without cardiovascular or renal comorbidities, and cardiovascular medications. In analyses adjusted for confounding factors, ARR was significantly associated with PWV but not with any other haemodynamic variable. The linear regression analyses confirmed that ARR was an independent explanatory factor for PWV, an acknowledged marker of large arterial stiffness^40,41^. As increased arterial stiffness is a strong independent predictor of cardiovascular events^41^, while higher ARR is associated with an increased incidence of cardiovascular disease^19^ and predicts future stroke in hypertensive patients^42^, a higher ARR may predispose to the future development of cardiovascular diseases.

Age is the most significant explanatory factor for large arterial stiffness^43,44^. In the present study, subjects in the highest ARR quartile were older than in the other quartiles. Consequently, the Figs. 1, 2 and 3 were adjusted for differences in age in addition to sex and estimated GFR. Moreover, despite the presence of age in the regression model, ARR was an independent explanatory factor for PWV. The inverse correlation between age and plasma renin is known from previous studies^45^. High sodium intake would also lower renin^1,5^, but this was not the cause for lower renin in the ARR quartiles 2–4 versus quartile 1, as 24-h sodium excretion to the urine was similar in all quartiles.

The screening positive cases with putative PA were excluded from our study population that was predominantly normotensive during the haemodynamic measurements. We found that ARR was directly and independently associated with large arterial stiffness, while the linear regression model displayed no direct association between serum aldosterone concentration or plasma renin activity and PWV. This finding may reflect higher renin-independent aldosterone release in the participants with high ARR. Indeed, a continuum of renin-independent aldosteronism in normotensive subjects has been previously identified^46,47^. High aldosterone levels related to prevailing plasma renin activity may predispose to the chronic adverse effects of aldosterone in the vascular system already prior to the clinical diagnosis of PA or hypertension^46^. The molecular basis for this continuum of aldosterone secretion may be explained by the aldosterone-producing cell clusters that have been discovered in the zona fasciculata of morphologically normal non-neoplastic adrenal glands: these clusters may be found in > 50% of normal adrenal glands, leading to mild autonomous aldosterone secretion and increased ARR with increasing age far more commonly than previously perceived^48,49^.

Some former studies have suggested a direct association between ARR and arterial stiffness. Shapiro et al. reported a significant association of ARR with PWV in 60 normotensive subjects, but in contrast to our study, their approach did not provide evidence of ARR as an independent explanatory factor of PWV^20^. In the Framingham study, Lieb et al. found that ARR was directly associated with PWV and four other measures of vascular pathophysiology^18^. As a major difference to our study, medicated hypertensive subjects and patients with known diabetes were not excluded from their analyses. Also, there was a 3-year interval between the laboratory examinations and haemodynamic measurements, whereas in our study these procedures were performed within a median period of 8 days from one another (25th to 75th percentile 2–17 days). A small study in 24 patients with essential hypertension by Mahmud and Feely found no correlation between ARR and PWV, but showed that aldosterone antagonist-induced decrease in systolic BP correlated with pre-treatment ARR^50^. However, in 102 patients with confirmed PA, no correlation was found between ARR and baseline PWV, or between ARR and the reduction in PWV following adrenalectomy^51^. Yet, the subsequent increase in plasma renin activity after adrenalectomy was an explanatory factor for the reduction in PWV during 6 months of follow-up^51^. Altogether, the effects of aldosterone excess on large arterial stiffness may be manifested early in the course of the disease, and the beneficial changes in the vasculature following surgical treatment do directly correlate with the plasma concentrations of aldosterone or ARR.

Noteworthy, in the present study the systolic and diastolic BP values were 13–16 and 16–17 mmHg lower, respectively, during the haemodynamic laboratory measurements than in the office measurements. This resembles the white-coat effect in hypertension, which has been related to large arterial stiffness in both untreated^52^ and treated hypertensive patients^53^. Out-of-office BP monitoring provides better assessment of overall BP and response to treatment in patients with white-coat hypertension^54^. The average difference between the office and laboratory BP in our study was rather large, whereby haemodynamic measurements under standard conditions can also result in clearly lower BP when compared with the office measurements. The correlations between office and laboratory measurements were 0.70 and 0.65 for systolic and diastolic BP, respectively. We used the algorithm recommended by The Endocrine Society for the screening of PA patients^1,5^, in order to exclude potential PA patients from the study population. Regardless of whether office BP > 150/100 mmHg measured on a single occasion, or average BP > 150/100 mmHg during haemodynamic measurements, was applied for the exclusion of participants, ARR was an independent explanatory factor for PWV. Of note, the results were corresponding when studied in normotensive and hypertensive subjects with two different cut-off values for hypertension (140/90 or 135/85 mmHg).

Seven of the present participants had fasting plasma glucose in the diabetic range, 53 participants had impaired fasting plasma glucose, while 89% of the participants presented with glucose values within the normal range. PA has been linked to abnormalities in glucose metabolism, especially to insulin resistance and impaired glucose tolerance^55–57^. Even in the absence of PA, plasma aldosterone concentration was related to insulin resistance in 251 male African American subjects^58^. Higher ARR was also associated with insulin resistance in 483 young adult African Americans without cardiovascular or renal disease^59^. In our study, no differences in insulin sensitivity between the ARR quartiles were observed as the indices QUICKI, HOMA-IR and Matsuda did not deviate. Therefore, the differences in PWV between the ARR quartiles could not be explained by variations in insulin sensitivity. Also the plasma concentrations of uric acid, the levels of which are usually elevated in insulin resistance and metabolic syndrome^23^, were similar in quartiles of ARR.

Changes in calcium metabolism may influence BP^60^, and induce alterations in the components of the renin-angiotensin system in the vasculature^61^. Moreover, vitamin D receptor activation downregulates the synthesis of renin in the juxtaglomerular cells and influences the expression of other components of the renin-angiotensin system in the kidney^62,63^. In the present study, the plasma concentrations of calcium and vitamin D metabolites were similar in the ARR quartiles. Therefore, putative changes in the metabolism of calcium and vitamin D were not the explanations for the differences in arterial stiffness between the ARR quartiles.

Our study has limitations. The cross-sectional design cannot substantiate causality. A potential selection bias caused by the recruitment of voluntary subjects and the exclusion protocol must be acknowledged. The haemodynamic recordings lasted for five minutes and the values of the last three minutes were used for the analyses, which gives a rather short window of observation. Still, the analyses were based on average on 190 cardiac cycles in each subject (mean HR in the study population was 63.3 beats/min). In addition, we applied indirect non-invasive methods requiring mathematical processing to derive PWV, stroke volume and cardiac output from the bioimpedance signal^36^, and central aortic BP waveform from the applanation tonometry signal^32^. Therefore, the results must be interpreted with caution, albeit the methods have been validated against direct or invasive measurements^31,35,37^. However, the approach to examine central haemodynamics instead of plainly focusing on brachial artery pressure, may be better related with the level of cardiovascular risk^64,65^.

In conclusion, a direct association between ARR and PWV was observed in 545 normotensive and never-treated hypertensive subjects when the screening criteria of PA were not met. Therefore, our results indicate that ARR is related to arterial stiffness in individuals without clinical suspicion of PA according to the prevailing guidelines. Altogether, increased ARR in the absence of stage II and more severe hypertension should be recognized as an indicator of increased cardiovascular risk.

## Data Availability

Analyses and generated datasets during the current study are not available publicly as our clinical database contains several indirect identifiers and the informed consent obtained does not allow publication of individual patient data. The datasets are available from the corresponding author on reasonable request.
